# *Taenia lynciscapreoli* in semi-domesticated reindeer (*Rangifer tarandus tarandus*, L.) in Sweden

**DOI:** 10.1016/j.ijppaw.2022.05.003

**Published:** 2022-05-06

**Authors:** Kautto Arja Helena, Grandi Giulio, Höglund Johan

**Affiliations:** a Swedish University of Agricultural Sciences, Department of Biomedical Sciences and Veterinary Public Health, Ulls Väg 26, Uppsala and Swedish Food Agency, Department of Control Support, Dag Hammarskjöldsväg 56 A, Uppsala, Sweden; b National Veterinary Institute, Department for Parasitology, Uppsala and Swedish University of Agricultural Sciences, Department of Biomedical Sciences and Veterinary Public Health, Section for Parasitology, Ulls Väg 26, Uppsala, Sweden; c Swedish University of Agricultural Sciences, Department of Biomedical Sciences and Veterinary Public Health, Section for Parasitology, Ulls Väg 26, Uppsala, Sweden

**Keywords:** *Taenia* spp., Metacestode, Reindeer, Lynx, Post mortem inspection, Sweden

## Abstract

We report here *Taenia lynciscapreoli* metacestode from the lung lobe of a semi-domesticated reindeer (*Rangifer tarandus tarandus*). The specimen was detected within a development project concerning remote post mortem inspection at a reindeer abattoir in Sweden. Post mortem inspection was performed according to a routine on-site official meat inspection protocol. The species identification to *T. lynciscapreoli* was confirmed based on the DNA extracted from the metacestode, which was analysed by sequencing of the cytochrome c oxidase subunit 1. Firstly, our finding shows that semi-domesticated reindeer in addition to several other cervids can act as an additional intermediate host for *T. lynciscapreoli*. Secondly, it further confirms that this parasite is more widely distributed on the Scandinavian peninsula than what has previously been shown. This is in line with a previous molecular finding of adult *T. lynciscapreoli* from the Eurasian lynx (*Lynx lynx*) in Sweden and demonstrates that new intermediate host can be detected. Whether the present finding can be regarded as accidental or have created opportunities for an expansion throughout the northernmost Scandinavian Peninsula remains to be seen.

## Highlights

• Semi-domesticated reindeer can act as the intermediate host for *T. lynciscapreoli.*
• *T. lynciscapreoli* is more widely distributed than previously shown.
• The impact on reindeer welfare remains unknown.

## 1 Introduction

The family Taeniidae includes among others genera *Taenia* and *Echinococcus* that have a global distribution, each of them including several important cestode species that infect a wide range of mammalian hosts. Zoonotic species in genus *Taenia* have received the most attention, especially since they still have a heavy impact on human health in developing countries (Murrell et al., 2005). Similarly, attention has also been placed on the genus *Echinococcus* which continues to be a global challenge (Wen et al., 2019). Even if they would occur mostly in wild canids and felids attention has to be placed on them because of the zoonotic implications, partly affecting the meat of intermediate hosts and partly their occurrence in domestic canids or felids living close to humans. Information on the host range and geographic distribution of taeniid parasites associated with reindeer and wild cervids is limited. Common to taeniids is that they have an indirect life cycle including two obligate mammalian hosts (Hoberg, 2006). Thus, parasite transmission, which occurs via carnivorism reflecting intimate predator-prey relationships, can include one or several definitive and intermediate host species, respectively (Schmidt and Roberts, 2009). The adult tapeworms in the small intestine of the definitive host can grow up to several meters long in case of *Taenia* whereas *Echinococcus* are only a few millimetres long. Depending on the species, the larval stages of both genera usually develop as a cysticercus or a modification thereof in several tissues and/or within the body cavity of their intermediate hosts (Jones and Pybus, 2001). Due to the potential public health issue, screening for metacestodes is part of the official post mortem inspection (PMI) of carcass and internal organs at slaughter (Anomynous, 2019). In European Union (EU) member states and in some third countries, the official routine PMI is performed according to the EU food safety legislation. Official control staff inspect on-site every carcass and offal visually. Palpation and incisions are done only in cases where abnormalities are observed. Official veterinarian shall declare meat infected with metacestodes unfit for human consumption (op.sit.). However, whenever the parasitic lesions are not disseminated across the whole carcass, non-infected tissues may be declared fit for human consumption after having undergone cold treatment. In PMI in commercial slaughter of all species of farmed and wild large game, the procedure dealing with cysticercos can be the same as for domestic Bovines and Suidae species. This official procedure is based on the precautionary principle and consent with the article 30, moreover, supported by article 45, point h and t in legislation (op.sit*.*). This approach is reasonable even if the article 30 can be assumed to deal only with *Taenia saginata and solium*.

There are at least three types of metacestodes relevant to semi-domesticated reindeer (*Rangifer t. tarandus*) in Fennoscandia. First, *Taenia hydatigena* typically forms cysticerci that are usually found in the omentum, mesentery, and peritoneum but rarely also in other nearby organs in a range of ungulates (Corda et al., 2020). Although *T. hydatigena* probably does not infect humans, infected organs should not be used to feed canids, because when the adult tapeworms grow, they can spread the infection within a reindeer population. Second, but less common is *Taenia krabbei* sensu lato, which can form a few millimeters long measle-like whitish cysts in the muscles of reindeer, but also in other cervids such as in moose (*Alces alces americanus*) (Lavikainen et al., 2011). The third type is *Echinococcus granulosus* sensu lato, which in contrast to *Taenia* spp. is characterized by the small size of the adult worms. Globally there are several strains or genotypes and they all have slightly different life cycles which some (G6, G8 and G10) are limited to the Northern hemisphere (Lavikainen et al., 2003; Oksanen and Lavikainen, 2015). The Nordic strains have the domestic dog (*Canis familiaris*) or wolf (*Canis lupus*) as definitive hosts, whereas reindeer or Eurasian moose serve as its intermediate host with metacestodes found predominately in the lungs, but rarely also in the liver. According to the European Zoonoses Reports (EFSA and ECDC, 2021a; EFSA and ECDC 2021b), the parasite is uncommon in reindeer today, but of major importance is that humans can function as an accidental intermediate host. Only occasional findings occurred in connection with PMI of reindeer during the 1990's (SVA, 1999). Nevertheless, it has been speculated that as the number of wolves increases, the occurrence of this parasite may increase, as it has in eastern Finland in the past (Hirvelä-Koski et al., 2003).

Moreover, there are several other taeniids that possibly can be transmitted between various carnivores and reindeer. According to Nakao et al. (2013), there are approximately 50 species recognised within the genus *Taenia*, among which many continue to be found as metacestodes in the Holarctic region. Lately, additional species in this genus have been described. One such example is *Taenia lynciscapreoli* which was described in 2016 from adult specimens isolated from the intestines of the Eurasian lynx (*Lynx lynx*) and the wolf (*Canis lupus*) in Finland and Russia (Haukisalmi et al., 2016; Lavikainen et al., 2013). The metacestodes have been detected mainly from the lungs of roe deer (*Capreolus capreolus*), but also from a few moose and Siberian roe deer (*Capreolus pygargus*) in Finland, Poland and/or Russia (Haukisalmi et al., 2016; Myczka et al., 2020). This indicates a south-eastern Eurasian distribution, which seems to rely most on predation of roe deer which is a preferred prey by lynx (Andrén and Liberg, 2015). Furthermore, *Taenia arctos* having a unique bear-moose cycle was described a decade ago in Finland (Haukisalmi et al., 2011). However, none of these *Taenia* spp*.* have so far been found in reindeer, despite the presence of expanding and overlapping populations with these large carnivores and reindeer in Fennoscandia during the last decades.

The number of reindeer in Sweden varies between 220 000 and 260 000 in winter herds and reindeer husbandry is taking place in the most northern parts of the country (Sametinget, 2022). The Swedish reindeer husbandry in the most northern parts of Sweden (even called Sápmi) is ethnic and is dependent on large grazing areas because the reindeer are herded over vast areas from the mountains towards the coast according to the seasons. Predation on reindeer is relatively common and sometimes a dominant component of the food base for the four large carnivores, i.e. brown bear (*Ursus arctos*), lynx, wolf and wolverine (*Gulo gulo*) (Bostedt and Grahn, 2008). Nevertheless, information on the host range and geographic distribution of taeniid parasites associated with reindeer and their predators is scarce both in Sweden and elsewhere. In this study, we report on a finding of a recently described tapeworm in the genus *Taenia* that mainly use the Eurasian lynx as its definitive host, but previously undescribed from reindeer.

## 2 Material and methods

### 2.1 Sample collection

The specimen described in this article was detected during a research project relating to development of official PMI with remote digital devices at commercial reindeer slaughter in Norrbotten County, Sweden. The research veterinarian was performing the PMI at a remote data screen whilst a technician at the slaughter line was filming the organs with a smartphone. The PMI was performed visually, and a whitish cyst was observed on the sagittal dorsal lobe surface of the right lung. The reindeer carcass, rest of the lungs and other organs were examined thoroughly by palpation and incisions. This female reindeer was in good condition (approximately three years old, weight 31.7 kg) and had been grazing in western mountains during the summer and in the forest region in eastern part of Norrbotten during the winter. The affected area was dissected and stored in formaline (10%). The specimen was submitted to the National Veterinary Institute (SVA) in Uppsala, Sweden, for diagnostic analysis, where after it was transferred to the Swedish University of Agricultural Sciences (SLU) for molecular diagnosis.

### 2.2 Sample preparation and sequencing

At SVA laboratorium, a cysticercus was found within the pulmonary cyst, photographed, and then rehydrated in distilled water for 2 day at room temperature. A portion of the neck of the cysticercus was dissected and used for molecular analyses. Genomic DNA was extracted at SLU using the Nucleospin Tissue Kit (Macherey-Nagel), following the guidelines issued by the manufacturer. The parasite species was identified following polymerase chain reaction (PCR) amplification of cytochrome c oxidase subunit 1 *(cox1)* region with the universal primers for taeniid species; JB3 and JP4.5 (Bowles et al., 1992). Observed bands from the PCR method were purified using the Illustra ExoProStar 1-step kit (VWR International, Stockholm, Sweden) and sent for Sanger sequencing (Macrogen, Amsterdam, The Netherlands). Sequencing results were analysed using Codon Code Aligner software (v. 7.1.2.) and then submitted for a nucleotide identity match using the Basic Local Alignment Search Tool (BLAST®) through the NCBI database (https://www.ncbi.nlm.nih.gov/guide/sequence-analysis/).

## 3 Results

### 3.1 Gross macroscopic finding

**Fig. 1:**
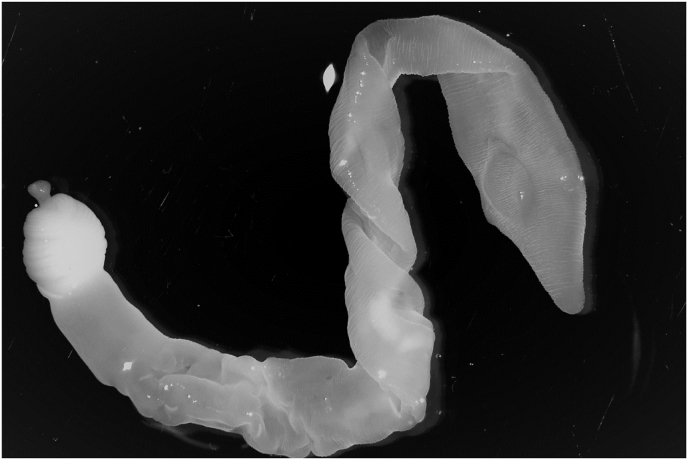
*Taenia lynciscapreoli*. Photo Anton de Jong, SVA.

A whitish cyst measuring around 2 cm protruded partially under the visceral pleura on sagittal position in the middle of the dorsal lobe surface of the right lung. Upon dissection a cysticercus (Fig. 1) was easily extracted; the metacestode itself was ≈4 cm long and was immersed in transparent, clear-opal fluid. Rest of the lungs and other organs as well as carcass were without pathological findings. No other cysts were found in the lungs.

### 3.2 Sequencing

The DNA, which was successfully amplified, matched the previously identified haplotypes of sequence data representing *T. lynciscapreoli*. The trimmed sequence (396 bp) was 100% identical with the *cox1* haplotype from a Eurasian lynx in Finland observed by Lavikainen et al. (2013) and by Myczka et al. (2020) in roe deer in Poland (GenBank accession numbers JX860629 and MK905226, respectively), whereas between 99.2% and 99.8% identical with those reported by Haukisalmi et al. (2016) in lynx (KU324546, KU324548), and wolves (KU324547) in Finland.

### 3.3 Data from PM inspection

According the SFA database commercial slaughter of reindeer has varied 2018–2021 between 45 000 and 55 000 reindeer per year. In 2018, two confirmed calcified cysticerci in cardiac muscle were found (45 500 reindeer slaughtered). After that, five findings were reported in 2021 (48 000 reindeer slaughtered): four calcified cysticerci in cardiac muscle and one living *Taenia lynciscapreoli* in a lung (the herein reported finding). In addition, between 2018 and 2021 game handling establishments in Sweden received yearly 15 000–19 000 fallow deer, about 6000 roe deer, 4400–5500 moose and 2500 red deer. No findings of cysticercosis were recorded in these cervids.

## 4 Discussion

Taeniid transmission is dependent on specific predator-prey relationships, however the life cycle of most species in wildlife hosts remain largely undescribed (Hoberg, 2002). This applies also to Sweden, where semi-domesticated reindeer and wild cervids carcasses are inspected for the presence of taeniid cysts at slaughter according to official PMI regulations in EU (Anomynous, 2019). Although metacestodes have been recorded previously in semi-domesticated or wild reindeer in Sweden, *T. lynciscapreoli* has never before been reported from reindeer.

To the best of our knowledge, so far only hydatid cysts of *E. granulosus* sensu lato have been reported from semi-domestic reindeer in Sweden. According to Ronéus (1974), and before unconditional compulsory official PMI for commercial reindeer slaughter, the infection rate was 1.6% out of nearly 1500 reindeer slaughtered from the most northern part of Sweden bounded by Finland and Norway. During winter 1996/1997 (SVA, 1999), three animals were diagnosed with cysts in the lungs (unpublished). Thus, the occurrence can be considered low today, which may be explained by the broken chain of transmission. As highlighted by Oksanen and Lavikainen (2015) herding dogs, which are proposed to be the most important definitive host for this parasite are today in few herds only complementary to scooters, motorbikes and helicopters throughout the reindeer husbandry area in Fennoscandia. There have also been successful information campaigns related to the associated risks to humans and the need of antiparasitic treatment of dogs living close to reindeer. Combined this seems to have resulted in only sporadic records. On the other hand, the population of wolves, which also serves as definitive hosts, has gradually increased during the last decades. Among 28 wolves shot in Sweden and examined by Lavikainen et al. (2011), only *T. hydatigena* and *T. krabbei* were found, but none were infected with *E. granulosus.* This is somewhat surprising as the parasite is known to be relatively common in wolves in Finland (Hirvelä-Koski et al., 2003). At the same time, all the investigated Swedish wolves came from the central parts of the country, outside the main reindeer herding area. Nevertheless, there is yet no evidence that the occurrence of *E. granulosus* has increased in response to an increased predator population.

Based on the typical appearance of the metacestode in the lungs as well as genetic information in the *cox1* region of the mitochondrial DNA, the present finding proved to be the recently described *T. lynciscapreoli* (Haukisalmi et al., 2016). Even though this species seems to have predominantly a lynx-roe deer cycle, it has been shown to be capable of infecting also wolves as definitive hosts, as well as Eurasian moose and Siberian roe deer as additional intermediate hosts (Lavikainen et al., 2013). As mentioned by Myczka et al. (2020), little is still known about the biology and life cycle of *T. lynciscapreoli.* Based on current knowledge *T. lynciscapreoli* has today a distribution in the south-west part of Finland, the northeast of Poland and in parts of Russia (*op. cit.*). However, it should be emphasized that it was described as late as 2016. Although there are no systematic studies on *Taenia* spp. in lynx available in Sweden, adult tapeworms are frequently found at necropsies of Eurasian lynx performed at SVA in connection with seasonal cullings. In 2021, 85% were infected with adult cestodes, and according to the molecular identification of a limited number of specimens, only *T. lynciscapreoli* has so far been identified (Maganira et al., 2019). The lynx population has recovered strongly since the 1990's when it became a protected species, and even if it has decreased somewhat recently, lynxes can still be found in most parts of Sweden. In 2022 the number of family groups (a female and her kitten/kittens) was estimated to be around 213 (Naturvardsverket, 2022). In addition, European roe deer have a more northern distribution today (Danilov et al., 2017), and are now also widespread and hunted in the coastal landscape in the northernmost parts of Sweden (Viltdata, 2022). It therefore seems reasonable to assume that the life cycle is maintained in the wild and thus reindeer run a risk of becoming infected when they migrate to overlapping grazing areas towards the coast during winter. Whether this will lead a further expansion and establishment of the cycle in northern Sweden, requires future investigation. However, in a study of lynx predation behaviour in Northern Sweden it was shown that semi-domestic reindeer contributed to over 90% of lynx ingested meat, calculated from both scats and kills (Pedersen et al., 1999).

## 5 Conclusions

The result of the DNA analysis in this study shows that the semi-domesticated reindeer (*Rangifer t. tarandus*) serves as a new intermediate host for *T. lynciscapreoli*. Together with other studies on the occurrence of *T. lynciscapreoli* in wildlife (Haukisalmi et al., 2016; Lavikainen et al., 2013; Myczka et al., 2020), it can be assumed that the lynx and roe deer are responsible for maintaining the parasite life cycle in south-central parts Sweden. However, our finding suggests that *T. lynciscapreoli* is spreading and expanding into areas with reindeer in more northern parts of the country. The impact on reindeer health and welfare and the role of the parasite as a potential source of infection for humans remains unknown.

## Declaration of competing interest

None.
